# Hypothyroidism and Type D Personality: Results From E-MPATHY, a Cross-sectional International Online Patient Survey

**DOI:** 10.1210/clinem/dgae140

**Published:** 2024-04-09

**Authors:** Petros Perros, Endre Vezekenyi Nagy, Enrico Papini, Juan Abad-Madroñero, Peter Lakwijk, Alan J Poots, Floortje Mols, Laszlo Hegedüs

**Affiliations:** Institute of Translational and Clinical Research, Newcastle University, Newcastle upon Tyne NE1 7RU, UK; Division of Endocrinology, Department of Medicine, Faculty of Medicine, University of Debrecen, Debrecen 4032, Hungary; Department of Endocrinology and Metabolism, Regina Apostolorum Hospital, Rome 00041, Italy; Data Science and Insight Team, Picker Institute Europe, Oxford, Oxfordshire OX4 2JY, UK; Thyroid Federation International, Transpolispark, Hoofddorp 2132 WT, The Netherlands; Data Science and Insight Team, Picker Institute Europe, Oxford, Oxfordshire OX4 2JY, UK; Department of Medical and Clinical Psychology, Tilburg University, Tilburg 5000 LE, The Netherlands; Department of Endocrinology, Odense University Hospital, Odense 5000, Denmark

**Keywords:** hypothyroidism, type D personality, questionnaire, survey

## Abstract

**Context:**

Between 10% and 15% of people with hypothyroidism experience persistent symptoms, despite achieving biochemical euthyroidism. The underlying causes are unclear. Type D personality (a vulnerability factor for general psychological distress) is associated with poor health status and symptom burden but has not been studied in people with hypothyroidism.

**Objective:**

To investigate type D personality in hypothyroidism and explore associations with other characteristics and patient-reported outcomes.

**Design:**

Multinational, cross-sectional survey.

**Setting:**

Online.

**Participants:**

Individuals with self-reported, treated hypothyroidism.

**Intervention:**

Questionnaire.

**Main Outcome Measures:**

Type D personality and associations with baseline characteristics, control of the symptoms of hypothyroidism by medication, satisfaction with care and treatment of hypothyroidism, impact of hypothyroidism on everyday living.

**Results:**

A total of 3915 responses were received, 3523 of which were valid. The prevalence of type D personality was 54.2%. Statistically significant associations were found between type D personality and several respondent characteristics (age, marital status, ethnicity, household income, comorbidities, type of treatment for hypothyroidism, most recent TSH level), anxiety, depression, somatization, poor control of the symptoms of hypothyroidism by medication, dissatisfaction with care and treatment of hypothyroidism, and a negative impact of hypothyroidism on everyday living).

**Discussion:**

Our study found a high prevalence of type D personality among people with hypothyroidism who responded to the survey. Type D personality may be an important determinant of dissatisfaction with treatment and care among people with hypothyroidism. Our findings require independent confirmation. Close collaboration between the disciplines of thyroidology and psychology is likely to be key in progressing our understanding in this area.

The prevalence of overt and subclinical hypothyroidism globally is reported to be 0.2% to 5.3% and 10%, respectively (1). Despite achievement of a serum TSH within the reference range, persistent symptoms occur in 10% to 15% of people with hypothyroidism (2). Hypotheses for the cause of these symptoms include (1) the inability of levothyroxine (L-T4) to emulate normal physiology and restore T3 levels in tissues; (2) confounding effects of comorbidities; (3) inflammation due to autoimmunity; (4) L-T4 prescribed or taken by patients suboptimally; (5) a high prevalence of unexplained symptoms, known as “somatic symptom disorder” (SSD) (2, 3); (6) being more likely to be investigated and diagnosed with minor, incidental perturbations of thyroid function (2); (7) the impact of the diagnostic label of having a chronic disease.

Type D personality has been linked to poor health and has been most often studied in patients with heart disease (4, 5). It is associated with persistent symptoms (6, 7-9), impaired quality of life (10, 11), mental health (11-14), treatment outcomes (11, 13-15), and adherence with medication (11, 13, 14, 16, 17). Type D personality is characterized by a predisposition to pessimism, worry, stress, negative affectivity (NA), and social inhibition (SI) (18, 19). The Type D Scale-14 (DS14) questionnaire, a self-administered and validated instrument, can be used to assess for type D personality (18). DS14 is subdivided into the subscales NA and SI. Individuals with high NA tend to experience negative emotions (such as feelings of dysphoria, anxiety, and irritability), have a negative view of themselves, and scan the world for signs of impending trouble. People with high SI tend to feel inhibited, tense, and insecure when with others (18). Individuals who display both high levels of NA and SI have a “distressed” or type D personality (18). Based on DS14 data, the prevalence of type D personality in the world population is estimated to be 21.0% to 38.5% (18, 20-22). It is found more frequently in primary care patients and in patients with a variety of morbidities (coronary heart disease, hypertension, chronic pain, asthma, tinnitus, sleep apnea, vulvovaginal candidiasis, mild traumatic brain injury, vertigo, melanoma, and diabetic foot syndrome) (18, 23). Studies of type D personality in people with thyroid disease are limited to survivors of thyroid cancer, where it did not predict quality of life or adherence with medication (24, 25).

In this study, type D personality in people with hypothyroidism was explored. The study questions were (1) what is the prevalence of Type D personality among people with hypothyroidism, and (2) what are the relationships between (2a) type D personality and respondent characteristics and (2b) type D personality and hypothyroidism-related patient-reported outcomes?

## Materials and Methods

### Study Design

E-MPATHY (E-Mode Patient self-Assessment of THYroid therapy) was a multinational, large-scale, cross-sectional, online study performed among people with a diagnosis of hypothyroidism from 68 countries (findings on patient satisfaction and SSD have been published) (3, 26).

### Survey Questionnaire

Survey development and delivery are described elsewhere (26). Briefly, the survey was cognitively tested across 5 rounds and translated from English into French, German, Italian, and Spanish by native speakers with idioms replaced. A pilot study of the English questionnaire preceded a full release for self-completion online between April 11, 2020, and January 3, 2021. Assessment and definitions of comorbidities are shown in Supplementary Table S1 (27). Potential participants were informed that the survey would take approximately 30 minutes to complete and were encouraged to complete it within 1 session. More than 1 response from the same IP address were automatically blocked.

### Personality, SSD, Anxiety, and Depression

#### DS14

The DS14 contains 2 subscales: NA and SI. The NA subscale measures the tendency to experience negative emotions (eg, anxiety, irritability, depressed mood), so people with high scores on this scale are likely to experience distress and dysphoria. The SI subscale assesses the tendency to inhibit self-expression in social interactions due to fear of disapproval. People with high scores may be reserved and introverted and avoid social interactions to prevent potential negative evaluations from others. The highest score for each subscale is 28 (18). We classified respondents using a 4-group categorization (24). Scores of ≥10 for each subscale are denoted by positive and <10 by negative signs; thus the 4 groups were (1) NA+ SI−, (2) NA− SI+, (3) NA+ SI+ (type D personality), and (4) NA− SI− (reference). A maximal attribution approach was taken: Where someone unequivocally scored more than 10 on a subscale, they were included as positive for that subscale, regardless of how many items they had answered. Similarly, if someone could not score more than 9 when accounting for any unanswered items in that scale, they were included as negative for that subscale. The English version of the DS14 was used, as well as translations into French, German, Italian, and Spanish performed by 2 certified native translators for each language for the purpose of our survey. Although validated French, German, Italian, and Spanish versions of the DS14 are available, these were not used in our study, as this was unknown to the coauthors responsible for designing the questionnaire at the time (P.P., L.H., E.V.N., E.P.). The data presented are for the full dataset (validated English and nonvalidated French, German, Italian, and Spanish translations). In addition to analyzing the full data (all languages, n = 3523), we examined the validated English-language responses (n = 2370) and the pooled nonvalidated translation French, German, Italian, and Spanish responses (n = 1153) and contrasted these with the full data.

#### SSD

SSD was assessed using the Patient Health Questionnaire-15 (PHQ-15), and a score of ≥10 on the PHQ-15 was considered as compatible with probable SSD (28). A maximal attribution approach was taken. All questions in the PHQ-15 specifically ask about symptoms experienced during the past 4 weeks.

#### Anxiety, low mood/depression

Participants were asked, “During the past 4 weeks, how much have you been bothered by anxiety?” and “During the past 4 weeks, how much have you been bothered by low mood/depression?” with the following response options: “bothered a little” or “bothered a lot” (considered as having anxiety or low mood/depression) and “not bothered at all” (considered as not having anxiety or low mood/depression). We used the method described previously (3) to create binarized variables for anxiety and low mood/depression. As for exploratory studies, the simplicity of interpretation with clear differentiation of categories offered by binarization provides a starting point to identify subsequent research questions.

### Patient-Reported Outcomes

#### Control of symptoms of hypothyroidism by medication

Participants responded to the statement “My hypothyroidism medication controls my symptoms well” with 6 response options on a Likert scale from “strongly disagree” to “strongly agree” and “uncertain.”

#### Satisfaction with treatment and care for hypothyroidism

Participants responded to “How satisfied are you with the overall care and treatment you have received for your hypothyroidism?” with 6 response options on a Likert scale from “very satisfied” to “very dissatisfied” and “don’t know.”

#### Impact of hypothyroidism on everyday living

Participants responded to the statement “My hypothyroidism has affected everyday activities that people my age usually do (eg, exercise, household chores, etc.)” with the 6 response options on a Likert scale from “strongly disagree” to “strongly agree” and “uncertain.”

### Dissemination of Questionnaire

Advertisements and information sheets to explain the purpose of the survey were prepared in the aforementioned 5 languages and promoted through Thyroid Federation International, a global network of patient thyroid disorder organizations, (https://thyroid-fed.org/) affiliates, and partners via social media and web pages.

### Inclusion Criteria

Participants self-identified as being more than 18 years old and using medication for hypothyroidism.

### Institutional Review Board Waiver Statement

The noninterventional nature of the survey and the fact that data were anonymous rendered the study exempt from institutional review board approval. The study was conducted in accordance with the Declaration of Helsinki as revised in 2013. All participants gave informed consent.

### Statistical Analyses

This study is a secondary analysis of a study (26), the data set of which was determined to be of sufficient size (>1066) to detect small to medium differences (delta 0.1) in statistics, with good confidence (95% power, alpha .05). The programming software Python 3.11 was used for statistical analyses.

We tested for association of the 4 type D personality groups with demographic and other baseline variables (sex, age, marital status, employment status, ethnic background, years of education, household income, comorbid conditions, current treatment for hypothyroidism, most recent TSH level, cause of hypothyroidism, self-reported anxiety, self-reported depression, PHQ-15 score) using chi-square tests, with a Bonferroni correction for multiple testing. The 4 type D personality groups were compared against the 3 patient-reported outcomes related to hypothyroidism (control of symptoms of hypothyroidism by medication, satisfaction with treatment and care for hypothyroidism, impact of hypothyroidism on everyday living). To explore what drove the associations, partial chi-square values (a standardization of the difference between observed and expected frequencies) were examined, as their size provides insight into the contribution to the chi-square statistic overall based on differences from the statistically expected data distributions.

Data are presented as percentages derived from the observed figures as a fraction of the total number of valid respondents (n = 3523), whereas chi-square data only included those cases where both type D personality status and the variable in question were known.

## Results

### Respondent Characteristics

**Figure 1.**
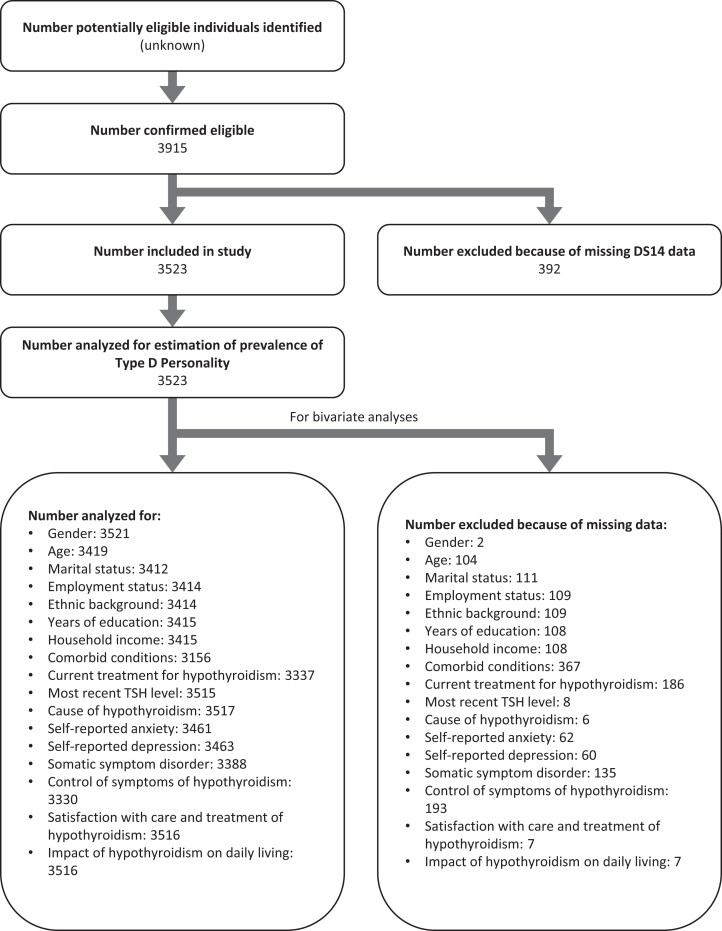
Flow chart showing included and excluded data. Abbreviations: DS14, Type D Scale-14; SSD, somatic symptom disorder.

A total of 3915 responses were received, 3523 of which contained valid DS14 data (90.0%) and comprised the study population (Table 1 and Fig. 1). Women comprised 94.3% (3323/3523) of respondents. Most respondents (72.6%; 2557/3523) were over 40 years old. Responses from the UK dominated (35.6%; 1255/3523). Most respondents were White (89.1%; 3145/3523), were employed (74.3%; 2619/3523), had received more than 8 years of education (87.1%; 3068/3523), had comorbidities (73.0%; 2573/3523), and were treated with L-T4 monotherapy (75.1%; 2645/3523). Time since diagnosis of hypothyroidism was <2 years in 10.1% (357/3523), 2 to 10 years in 36.4% (1283/3523), >10 years in 51.2% (1805/3523), and missing data or “don’t know” in 2.2% (78/3523). The time between the last serum TSH check and completing the questionnaire was within the last 2 months in 45.1% (1589/3523), >2 to 6 months in 27.9% (983/3523), >6 to 12 months in 16.0% (565/3523), >12 months in 10.2% (358/3523), and missing data or “don’t know/can’t remember” in 0.8% (28/3523).

**Table 1.** Baseline respondent characteristics

	All respondents		Respondents by type D personality group							
			NA+ SI−		NA− SI+		NA+ SI+ (type D personality)		NA−SI− (reference)	
	n	% (n 3523)	n	%	n	%	n	%	n	%
Number of respondents	3523	100.0	735	20.9	299	8.5	1908	54.2	581	16.5
Sex										
Male	169	4.8	27	0.8	22	0.6	89	2.5	31	0.9
Female	3323	94.3	701	19.9	275	7.8	1802	51.2	545	15.5
Prefer not to say/prefer to self-identify	29	0.9	7	0.2	1	0.0	16	0.5	5	0.1
Missing sex data	2	0.0	0	0.0	1	0.0	1	0.0	0	0.0
Age (years)										
18-30	241	6.8	42	1.2	21	0.6	157	4.5	21	0.6
31-40	621	17.6	120	3.4	56	1.6	371	10.5	74	2.1
41-50	968	27.5	201	5.7	79	2.2	536	15.2	152	4.3
51-60	879	25.0	183	5.2	68	1.9	466	13.2	162	4.7
61-70	559	15.9	121	3.4	53	1.5	270	7.7	115	3.3
≥71	151	4.3	38	1.1	18	0.5	58	1.6	37	1.1
Missing data	104	2.9	30	0.9	4	0.1	50	1.4	20	0.6
Top 10 countries by respondent number										
United Kingdom	1255	35.6	234	6.6	86	2.4	750	21.3	185	5.3
France	595	16.9	142	4.0	35	1.0	335	9.5	83	2.4
Sweden	194	5.5	32	0.9	21	0.6	107	3.0	34	1.0
Finland	147	4.2	25	0.7	25	0.7	73	2.1	24	0.7
Australia	140	4.0	26	0.7	16	0.5	71	2.0	27	0.8
Italy	122	3.5	35	1.0	7	0.2	55	1.6	25	0.7
Germany	110	3.1	16	0.5	17	0.5	51	1.4	26	0.7
Norway	99	2.8	22	0.6	15	0.4	37	1.1	25	0.7
USA	102	2.9	22	0.6	11	0.3	51	1.4	18	0.5
Canada	106	3.0	22	0.6	15	0.4	49	1.4	20	0.6
Other	498	14.1	120	3.4	43	1.2	249	7.1	86	2.4
Missing country data	155	4.4	39	1.1	8	0.2	80	2.3	28	0.8
Marital status										
Married/partnership	2402	68.2	530	15.0	188	5.3	1274	36.2	410	11.6
Single/divorced/widowed	911	25.9	155	4.4	102	2.9	517	14.7	137	3.9
Prefer not to say	59	1.7	10	0.3	4	0.1	35	1.0	10	0.3
Other	40	1.1	10	0.3	1	0.0	28	0.8	1	0.0
Missing marital statsus data	111	3.1	30	0.9	4	0.1	54	1.5	23	0.7
Employment status										
Working (full-time, part-time, student, carer)	2619	74.3	545	15.5	236	6.7	1409	40.0	429	12.2
Not working	602	17.1	119	3.4	46	1.3	347	9.8	90	2.6
Prefer not to say	58	1.6	9	0.3	7	0.2	31	0.9	11	0.3
Other	135	3.8	30	0.9	6	0.2	69	2.0	30	0.9
Missing employment data	109	3.1	32	0.9	4	0.1	52	1.5	21	0.6
Ethnic background										
White	3145	89.1	641	18.2	270	7.7	1701	48.3	533	15.1
Other	218	6.2	56	1.6	25	0.7	119	3.4	18	0.5
Prefer not to say	51	1.4	8	0.2	0	0.0	35	1.0	8	0.2
Missing ethnic data	109	3.1	30	0.9	4	0.1	53	1.5	22	0.6
Years of education										
8 years or less	272	7.7	69	2.0	12	0.3	158	4.5	33	0.9
More than 8 years	3068	87.1	622	17.7	278	7.9	1653	46.9	515	14.6
Prefer not to say	75	2.1	14	0.4	5	0.1	45	1.3	11	0.3
Missing education data	108	3.1	30	0.9	4	0.1	52	1.5	22	0.6
Household income										
Above average	1081	30.7	230	6.5	122	3.5	491	13.9	238	6.8
Average	1534	43.5	324	9.2	110	3.1	874	24.8	226	6.4
Below average	634	18.0	115	3.3	51	1.4	399	11.3	69	2.0
Prefer not to say	124	1.2	26	0.7	10	0.3	65	1.8	23	0.7
Don't know	42	1.2	9	0.3	2	0.1	27	0.8	4	0.1
Missing household income data	108	3.1	31	0.9	4	0.1	52	1.5	21	0.6
Comorbidities										
No comorbid conditions	583	16.5	113	3.2	69	2.0	286	8.1	115	3.3
Mental illness	858	24.4	179	5.1	30	0.9	599	17.0	50	1.4
Other comorbid conditions	1715	48.7	371	10.5	165	4.7	836	23.7	343	9.7
Missing comorbidity data	367	10.4	72	2.0	35	1.0	187	5.3	73	2.1
Median (n range)	2 (0-12)		2 (0-10)		1 (0-9)		2 (0-12)		1 (0-9)	
Current treatment for hypothyroidism										
L-T4	2645	75.1	560	15.9	213	6.0	1472	41.8	400	11.4
L-T3	73	2.1	17	0.5	7	0.2	39	1.1	10	0.3
DTE	265	7.5	50	1.4	40	1.1	121	3.4	54	1.5
L-T4 + L-T3	354	10.0	59	1.7	33	0.9	174	4.9	88	2.5
Missing treatment data	186	5.3	49	1.4	6	0.2	102	2.9	29	0.8
Most recent TSH level										
Normal	1232	35.0	276	7.8	92	2.6	671	19.0	193	5.5
High	410	11.6	84	2.4	24	0.7	231	6.6	71	2.0
Low	1076	30.5	218	6.2	121	3.4	535	15.2	202	5.7
Don't know/can't remember	797	22.6	156	4.4	62	1.8	467	13.3	112	3.2
Missing TSH data	8	0.2	1	0.0	0	0.0	4	0.1	3	0.1
Cause of hypothyroidism										
Hashimoto/autoimmune disease	1285	36.5	261	7.4	126	3.6	678	19.2	220	6.2
Treatment for Graves' disease or hyperthyroidism	289	8.2	47	1.3	24	0.7	167	4.7	51	1.4
Treatment for thyroid cancer	451	12.8	104	3.0	37	1.1	223	6.3	87	2.5
Treatment for benign goiter	174	4.9	40	1.1	6	0.2	97	2.8	31	0.9
Pregnancy related	133	3.8	34	1.0	7	0.2	75	2.1	17	0.5
Other	1185	33.6	246	7.0	99	2.8	665	18.9	175	5.0
Missing data	6	0.2	3	0.1	0	0.0	3	0.1	0	0.0
Self-reported anxiety										
No	1164	33.0	188	5.3	185	5.3	426	12.1	365	10.4
Yes	2297	65.2	536	15.2	110	3.1	1452	41.2	199	5.6
Missing anxiety data	62	1.8	11	0.3	4	0.1	30	0.9	17	0.5
Self-reported depression										
No	1076	30.5	180	5.1	183	5.2	345	9.8	368	10.4
Yes	2387	67.8	543	15.4	114	3.2	1532	43.5	198	5.6
Missing data	60	1.7	12	0.3	2	0.1	31	0.9	15	0.4
Somatic symptom disorder (PHQ-15 score)										
<10	1406	39.9	286	8.1	176	5.0	588	16.7	356	10.1
≥10	1982	56.3	421	12.0	108	3.1	1261	35.8	192	5.4
Missing PHQ-15 data	135	3.8	28	0.8	15	0.4	59	1.7	33	0.9

Abbreviations: DTE, desiccated thyroid extract; L-T3, triiodothyronine; L-T4 + L-T3, combination treatment with levothyroxine (L-T4) and triiodothyronine (L-T3); L-T4, levothyroxine; NA, negative affectivity; PHQ-15, Patient Health Questionnaire 15; SI, social inhibition.

The 4 type D personality groups are shown as defined by the subscales NA and SI (NA+ SI−, NA− SI+, NA+ SI+ (type D personality) and NA− SI− (reference), where + denotes a score ≥10 and − a score of <10.

### Type D Personality

The data were divided into the 4 personality groups as defined by the subscales NA and SI; NA+ SI−, NA− SI+, NA+ SI+ (type D personality), and NA− SI− (reference). By this definition, the prevalence of type D personality in this cohort of people with hypothyroidism was NA+ SI− 20.9% (735/3523), NA−SI+ 8.5% (299/3523), NA+SI+ (type D) 54.2% (1908/3523), NA−SI− (reference) 16.5% (581/3523).

#### Associations with respondent characteristics

Statistically significant associations were found between type D personality and age, marital status, ethnicity, household income, comorbidities, type of treatment for hypothyroidism, most recent TSH levels, anxiety, depression, and SSD (Table 2). With a 4-category outcome and multinominal independent variables, describing the directionality of the associations has challenges. One way to consider how an association is detected is to examine the “partial chi-squares” (Supplementary Table S2) (27). Partial chi-squares represent the contribution of the particular combination of factors. The larger the partial, the greater the contribution to any detected association. The largest contributions to the distribution were most commonly with the reference group (NA−SI−, not having type D personality) and effects arising due to youngest (age 18-40 years) and oldest (age >61 years), being single/divorced, non-White ethnicity, above and below average income, low TSH, desiccated thyroid extract (DTE) and L-T4 + L-T3 treatment, comorbidity, no anxiety, and no depression (Supplementary Fig. S1) (27).

**Table 2.** Chi-square analysis for observed variables against the 4 type D personality groups as defined by the subscales NA and SI

		NA+ SI−		NA− SI+		NA+ SI+ (type D personality)		NA− SI− (reference)		Chi	*P*	Adjusted significance
		O	E	O	E	O	E	O	E			
Sex										7.77	2.56E-01	Not significant
	Male	27	35.3	22	14.3	89	91.5	31	27.9			
	Female	701	693.7	275	281.2	1802	1799.8	545	548.3			
	Prefer not to say/Prefer to self-identify	7	6.1	1	2.5	16	15.7	5	4.8			
Age (years)										57.60	6.49E-07	Significant
	18-30 years	42	49.7	21	20.8	157	131.0	21	39.5			
	31-40 years	120	128.1	56	53.6	371	337.5	74	101.9			
	41-50 years	201	199.6	79	83.5	536	526.0	152	158.8			
	51-60 years	183	181.3	68	75.8	466	477.7	162	144.2			
	61-70 years	121	115.3	53	48.2	270	303.8	115	91.7			
	71 years or over	38	31.1	18	13.0	58	82.1	37	24.8			
Marital status										29.84	4.68E-04	Significant
	Married/partner	530	496.3	188	207.7	1274	1305.2	410	392.8			
	Single/divorced	155	188.2	102	78.8	517	495.0	137	149.0			
	Prefer not to say	10	12.2	4	5.1	35	32.1	10	9.6			
	Other	10	8.3	1	3.5	28	21.7	1	6.5			
Employment status										11.22	2.61E-01	Not significant
	Working (full-time, part-time, student, carer)	545	539.3	236	226.3	1409	1423.8	429	429.6			
	Not working	119	124.0	46	52.0	347	327.3	90	98.7			
	Prefer not to say	9	11.9	7	5.0	31	31.5	11	9.5			
	Other (please specify)	30	27.8	6	11.7	69	73.4	30	22.1			
Ethnic background										21.21	1.68E-03	Significant
	White	641	649.5	270	271.8	1701	1708.8	533	515.0			
	Other	56	45.0	25	18.8	119	118.5	18	35.7			
	Prefer not to say	8	10.5	0	4.4	35	27.7	8	8.4			
Years of education										14.59	2.37E-02	Not significant
	8 years or less	69	56.2	12	23.5	158	147.8	33	44.5			
	More than 8 years	622	633.4	278	265.0	1653	1667.4	515	502.2			
	Prefer not to say	14	15.5	5	6.5	45	40.8	11	12.3			
Household income										79.93	4.26E-12	Significant
	Above average	230	222.8	122	93.4	491	587.5	238	177.3			
	Average	324	316.2	110	132.5	874	833.7	226	251.5			
	Below average	115	130.7	51	54.8	399	344.6	69	104.0			
	Prefer not to say	26	25.6	10	10.7	65	67.4	23	20.3			
	Don't know	9	8.7	2	3.6	27	22.8	4	6.9			
Comorbid conditions										164.69	6.01E-33	Significant
	No comorbid conditions	113	122.5	69	48.8	286	317.9	115	93.8			
	Mental illness	179	180.2	30	71.8 70.7	599	467.9	50	138.1			
	Other comorbid conditions	371	360.3	165	143.5	836	935.2	343	276.1			
Current treat-ment for hypo-thyroidism										44.5	1.13E-06	Significant
	L-T4	560	543.7	213	232.2	1472	1431.5	400	437.5			
	L-T3	17	15.0	7	6.4	39	39.5	10	12.1			
	DTE	50	54.5	40	23.3	121	143.4	54	43.8			
	L-T4 + L-T3	59	72.8	33	31.1	174	191.6	88	58.6			
Most recent TSH level										31.34	2.59E-04	Significant
	Normal	276	257.3	92	104.8	671	667.3	193	202.6			
	High	84	85.6	24	34.9	231	222.1	71	67.4			
	Low	218	224.7	121	91.5	535	582.8	202	176.9			
	Don't know/can't remember	156	166.4	62	67.8	467	431.7	112	131.1			
Cause of hypothyroidism										25.6	4.19E-02	Not significant
	Hashimoto/autoimmune disease	261	267.4	126	109.2	678	696.0	220	212.3			
	Treatment for Graves' or hyperthyroidism	47	60.2	24	24.6	167	156.5	51	47.7			
	Treatment for thyroid cancer	104	93.9	37	38.3	223	244.3	87	74.5			
	Other	246	246.6	99	100.7	665	641.9	175	195.8			
	Treatment for benign goiter	40	36.2	6	14.8	97	94.2	31	28.7			
	Pregnancy related	34	27.7	7	11.3	75	72.0	17	22.0			
Self-reported anxiety										475.8	8.30E-103	Significant
	No	188	243.5	185	99.2	426	631.6	365	189.7			
	Yes	536	480.5	110	195.8	1452	1246.4	199	374.3			
Self-reported depression										587.9	4.13E-127	Significant
	No	180	224.6	183	92.3	345	583.2	368	175.9			
	Yes	543	498.4	114	204.7	1532	1293.8	198	390.1			
Somatic symptom disorder (PHQ-15 score)										245.26	6.93E-53	Significant
	PHQ <10	286	293.4	176	117.9	588	767.3	356	227.4			
	PHQ ≥10	421	413.6	108	166.1	1261	1081.7	192	320.6			

Abbreviations: DTE, desiccated thyroid extract; E, expected values; L-T3, triiodothyronine; L-T4 + L-T3, combination treatment with levothyroxine (L-T4) and triiodothyronine (L-T3); L-T4, levothyroxine; NA, negative affectivity; O, observed values; PHQ-15, Patient Health Questionnaire 15; SI, social inhibition.

The 4 type D personality groups are shown as defined by the subscales NA and SI (NA+ SI−, NA− SI+, NA+ SI+ (type D personality) and NA− SI− (reference), where + denotes a score ≥10 and − a score of <10.

The adjusted threshold by Bonferroni method was for the *P*-level of .002294. The table includes all data (both English and non-English translations of the Type D Scale-14). The column labeled “adjusted significance” is derived after Bonferroni correction.

The association with SSD was probably driven by divergences in the expected numbers in the reference group for type D personality (NA−SI−), also suggested by the prevalence of SSD (PHQ-15 score ≥10) in the 4 groups of type D, which was highest in the NA+SI (type D personality) and lowest in the NA−SI− (reference group): NA+SI− 57.3% (421/735), NA−SI+ 36.1% (108/299), NA+SI+ (type D personality) 66.1% (1261/1908), and NA−SI− (reference) 33.0% (192/581).

No associations were found between type D personality and sex, cause of hypothyroidism, years of education, or employment status.

#### Associations with patient-reported outcomes

**Figure 2.**
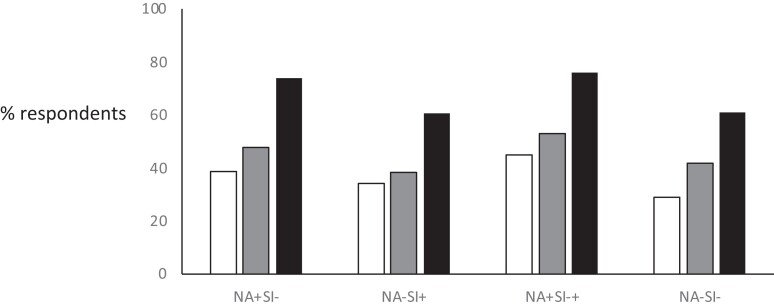
Patient reported outcomes by type D personality groups. White columns show percentage of respondents who indicated that their thyroid medication controlled symptoms of hypothyroidism poorly (sum of “tend to disagree” and “strongly disagree” in response to the statement “my hypothyroidism medication controls my symptoms well”); grey columns show percentage of respondents who indicated that they were dissatisfied with their care and treatment for hypothyroidism (sum of “slightly dissatisfied” and “very dissatisfied” in response to question “how satisfied are you with the overall care and treatment you have received for your hypothyroidism?”); black columns show percentage of respondents who indicated that hypothyroidism had a negative impact on everyday living (sum of “tend to agree” and “strongly agree” in response to the statement “my hypothyroidism has affected everyday activities that people my age usually do eg, exercise, household chores, etc.”). The largest differences noted were between groups NA+SI+ (type D personality) and NA−SI− (reference). The percentages were calculated as follows: white columns “tend to disagree” + “strongly disagree”/“tend to disagree” + “strongly disagree” + “tend to agree” + “strongly agree” + “neither agree nor disagree”; grey columns: “slightly dissatisfied” + “very dissatisfied”/“slightly dissatisfied” + “very dissatisfied” + “slightly satisfied” + “very satisfied” + “neither agree nor disagree”; black columns “tend to agree” + “strongly agree”/“tend to agree” + “strongly agree” + “tend to disagree” + “strongly disagree” + “nether agree nor disagree”. Missing data and “don’t know” (for grey columns), “don’t know/can’t recall,” and “this does not apply to me” (for black columns) and “missing data” (for all columns) were excluded from the calculations. Abbreviations: NA, negative affectivity; SI, social inhibition.

Type D personality was associated with the expression of the view that the thyroid medication taken did not control the symptoms of hypothyroidism well (*P* = 6.32E-24), with dissatisfaction with care and treatment of hypothyroidism (*P* = 1.45E-18) and with a negative impact on everyday living (*P* = 5.00E-29) (Table 3, Fig. 2). Partial chi-squares suggest that NA largely drove these associations (Supplementary Table S2) (27).

**Table 3.** Associations between type D personality and patient-reported outcomes

		NA+ SI−		NA− SI+		NA+ SI+ (type D personality)		NA− SI− (reference)		Chi	*P*	Adjusted significance
		O	E	O	E	O	E	O	E			
Symptoms of hypothyroidism are controlled by medication										139.9	6.32E-24	Significant
	Strongly agree	102	105.3	64	44.7	189	277.2	157	84.7			
	Tend to agree	187	194.2	88	82.5	505	511.1	164	156.2			
	Neither agree nor disagree	132	111.5	40	47.4	300	293.5	70	89.7			
	Tend to disagree	167	170.1	65	72.3	494	447.8	101	136.8			
	Strongly disagree	97	103.9	34	44.1	315	273.4	59	83.6			
Satisfaction with overall care and treatment for hypothyroidism										120.59	1.45E-18	Significant
	Very satisfied	105	102.9	66	41.9	186	266.8	136	81.3			
	Slightly satisfied	168	166.4	80	67.8	403	431.4	146	131.5			
	Neither satisfied nor dissatisfied	106	102.5	38	41.8	294	265.7	53	81.0			
	Slightly dissatisfied	157	143.6	48	58.5	383	372.4	100	113.5			
	Very dissatisfied	190	211.9	66	86.3	618	549.4	141	167.4			
	Don't know	8	6.7	1	2.7	19	17.3	4	5.3			
Hypothyroidism has affected everyday activities negatively										181.43	5.00E-29	Significant
	Strongly agree	305	299.8	78	122.3	879	779.1	176	236.8			
	Tend to agree	211	207.9	91	84.8	550	540.2	145	164.2			
	Neither agree nor disagree	78	73.6	30	30.0	185	191.3	60	58.1			
	Tend to disagree	54	53.0	26	21.6	119	137.6	55	41.8			
	Strongly disagree	50	63.0	54	25.7	100	163.6	98	49.7			
	Don't know/can't recall	13	13.8	6	5.6	33	35.8	14	10.9			
	This does not apply to me	22	22.1	14	9.0	39	57.4	31	17.5			

Abbreviations: E, expected values; NA, negative affectivity; O, observed values; SI, social inhibition.

Chi-square analysis for hypothyroid patient reported outcomes against the 4 type D personality groups as defined by the subscales NA and SI (NA+ SI−, NA− SI+, NA+ SI+ (Type D personality) and NA− SI− (reference), where + denotes a score ≥10 and − a score of <10. The adjusted threshold by Bonferroni method was for the *P*-level of .002294. The table includes all data (both English and non-English translations of Type D Scale-14). The column labeled “adjusted significance” is derived after Bonferroni correction.

### English and Non-English Translations of DS14

The results presented were derived from all respondents using the validated English version of the DS14, as well as translations into French, German, Italian, and Spanish performed by 2 certified native translators for each language. The analyses for type D personality were repeated for respondents using data for the English and non-English language translations (Supplementary Table S3) (27). The concordance in the *P*-values between the analyses from all respondents and English-language respondents was high with only 1 (ethnic background) of 17 variables discordant. The discordance between all respondents and non-English language respondents was slightly greater (4 of 17; age, marital status, ethnicity, and most TSH), with similar results for English vs non-English language respondents (3 of 17, age, marital status, and most recent TSH).

## Discussion

Type D personality (NA+SI+) is common, and it is associated with persistent physical symptoms and anxiety and depressive symptoms (13). While impaired quality of life and dissatisfaction with care and treatment are well documented in hypothyroidism (29), data on type D personality are confined to 2 studies in thyroid cancer survivors, showing no association with quality of life or adherence with medication (24, 25). In our study, we provide a starting point that highlights areas for exploration.

We used the DS14 (18) to assess type D personality and to test the hypothesis that type D personality may be associated with clinical characteristics and patient-reported outcomes related to hypothyroidism. Respondents' characteristics were similar to people with hypothyroidism reported in the literature (1, 30, 31).

### Type D Personality (NA+SI+)

The proportion of respondents with type D personality (NA+SI+) was higher (54.2%) than the reported prevalence in the general population (21-38.5%) (18, 20-22) and that reported in patients with heart disease (21-35%) (32), skin diseases (39-43%) (33), and obstructive sleep apnea (32.5%) (34) but similar to that reported for diabetes (52%) (35). It was higher than the 20.8% prevalence reported among survivors of thyroid cancer (25). Type D personality is common among patients with chronic diseases (36); thus it is not surprising there is a high prevalence in people with hypothyroidism, though selection bias may have inflated this figure. Both behavioral and biological mechanisms including common genetic predispositions that implicate type D personality in disease causation have been proposed as potential explanations of the association between type D personality and disease (19).

#### Type D personality and respondent characteristics

In our study, type D personality traits were associated with several respondent characteristics (clear drivers as revealed by the partial chi-square values were youngest and oldest ages, ethnicity other than White, having above average household income, having mental illness, treatment with DTE and L-T4 + L-T3, having a low most recent TSH level, having no anxiety, and having no depression and not having SSD). The trend noted for clustering of treatment with DTE or L-T4 + L-T3 and non-type D personality is interesting. Given that type D personality traits are stable across time (37), it is unlikely that treatment with DTE or L-T4 + L-T3 would reverse those features. Alternatively, people with hypothyroidism and non-type D personality may be more capable of accessing DTE or L-T4 + L-T3 treatment because they are less socially inhibited. The association of type D personality with a low TSH is interesting and potentially related to the use of DTE or L-T4 + L-T3 often resulting in overtreatment (38). In another study, we showed that probable SSD is highly prevalent in the same cohort of patients (3). An association between SSD and type D personality has been reported in people without hypothyroidism in the past (13) and is now noted in people with hypothyroidism.

#### Type D personality and patient-reported outcomes

Type D personality was associated with poor control of symptoms of hypothyroidism by medication, dissatisfaction with overall treatment and care for hypothyroidism, and a negative impact of hypothyroidism on everyday living. These associations are probably driven by NA. Our findings are consistent with other studies showing an association of type D personality with negative outcomes and that this effect is mainly driven by NA (39-41). This may be related to NA predisposing to chronic stress and physiological and immunological responses, unhealthy behaviors as coping mechanisms, and adverse effects on social relationships and support systems. The experience of somatic symptoms in somatization has been associated with harm avoidance and NA (42). Compared to people without somatization, people with somatization show more self-defeating, depressive, and passive-aggressive personality traits and neuroticism and less agreeableness and extraversion (43).

### English and Non-English Translations of the DS14

There were only a few differences in the results of the analyses between English and non-English language users; thus the fact that the non-English translations were not independently validated did not appear to impact on our findings. Ethnic background was the only respondent characteristic that was significant in the full data but not statistically significant in the validated English translation data.

### Significance of Main Findings

Hypothyroidism is known to be associated with psychological morbidity both before and after the diagnosis of hypothyroidism (44). Our study indicates that type D personality may cluster with hypothyroidism. Given that the majority of patients develop hypothyroidism in middle life, it may be assumed that type D personality precedes the onset of hypothyroidism in most cases. This reasoning suggests that the type D personality traits are not the result of hypothyroidism or its treatment and the poor experiences often described by people with hypothyroidism but that the pre-existence of type D personality traits may color the perception of the patient experience.

The significance of our findings rests with how clinicians approach the common scenario of people with hypothyroidism and persistent unexplained symptoms. Research in Europe conducted between 2019 and 2021 (45, 46) and in Latin America in 2022 (47) shows that thyroid specialists usually offer pharmacological solutions to such patients in the form of combination therapy of L-T4 + L-T3, despite evidence from randomized controlled studies indicating no benefit from combination treatment compared to L-T4 alone (29).

A study of people without hypothyroidism with a high prevalence of type D personality, SSD, anxiety, and depression (13) showed that psychological interventions were associated with improvements in anxiety, depression, and physical symptoms (13). Thus, psychological therapies may be appropriate for some people with hypothyroidism, particularly those with type D personality and SSD, who can be identified by using the validated questionnaires DS14 and PHQ-15.

### Limitations

The study has limitations. Some nations were overrepresented, there were very few men, and the demographic characteristics and medical background in our sample could limit generalizability of the results. The sample may not represent the population of people with hypothyroidism since respondents were invited via patient organizations and social media and there was some sample heterogeneity. The diagnosis of hypothyroidism was not validated independently, and it is possible that some patients may have been treated for hypothyroidism despite normal thyroid biochemistry. In addition, the assessments of satisfaction, control of symptoms of hypothyroidism by medication, impact on daily life, and anxiety and depression did not utilize validated instruments. Data on whether depression was treated or not were not available. The survey was conducted during the COVID pandemic, which may have influenced responses. The results of our study needs to be considered in the context of the fact that dissatisfied patients are more likely to respond to surveys (26, 48, 49). We used univariate analyses. The significant findings reported here may not persist after multivariate analyses. However, our aims were exploratory, and our findings are of interest themselves, providing useful information not previously available. In mitigation of these factors, the sample size (which was well beyond that specified by power calculations), cognitive testing, piloting, and inclusion of a patient representative in the research team were strengths.

## Conclusions

This study demonstrates a high prevalence of type D personality among people with hypothyroidism who responded to the survey. There were associations between type D personality, several respondent characteristics, somatization, and negative patient-reported outcomes regarding hypothyroidism in an adequately powered sample. Type D personality may be an important determinant of dissatisfaction with treatment and care among some people with hypothyroidism. Our findings require independent confirmation from studies that focus on type D personality. Close collaboration between the disciplines of thyroidology and psychology is likely to be key in progressing our understanding in this area.

## Data Availability

All datasets generated during and/or analyzed during the current study are not publicly available but are available from the corresponding author on reasonable request.

## Abbreviations

DTE: desiccated thyroid extract
L-T3: triiodothyronine
L-T4: levothyroxine
NA: negative affectivity
PHQ-15: Patient Health Questionnaire-15
SI: social inhibition
SSD: somatic symptom disorder
